# Refugee mothers’ mental health in Denmark: possibilities and limits of home visiting programs

**DOI:** 10.1007/s00737-024-01529-y

**Published:** 2024-11-19

**Authors:** Maria Marti-Castaner, Vivian Rueskov Poulsen, Ezio Di Nucci, Sarah Fredsted Villadsen

**Affiliations:** 1 Department of Public Health, Section of Health Services Research, University of Copenhagen, Øster Farimagsgade 5, Room 15.0.11, Copenhagen, DK-1014 Denmark; 2 Department of Public, Health, Section of Social Medicine, University of Copenhagen, Copenhagen, Denmark

**Keywords:** Refugee, Mothers, Perinatal, Nurse home visiting, Mental health, Well-being, Social support, Integration, Migration

## Abstract

**Purpose:**

To explore the experiences of refugee mothers and community health nurses participating in a nurse home visiting program in Denmark, focusing on the program’s effects on the psychosocial well-being of refugee mothers during the transition to motherhood.

**Methods:**

The nurse home visiting program was an add-on the public care offered to all families, with extra training of the community health nurses and more time to engage with the families with immigrant and refugee backgrounds. Community health nurses (12) and participating women (9) participated in qualitative interviews between September and December 2020, following the program’s conclusion.

**Results:**

Utilizing the Resource-Based Model of refugee adaptation as a theoretical framework, we identified four main themes: (i) negotiating parenting norms and gaining confidence through parenting resources; (ii) finding emotional support to cope with integration pressures; (iii) expanding social resources, (iv) building bridges with welfare state services. These themes captured the resources gained by mothers through the home visiting program, positively influencing their psychosocial well-being, while also acknowledging the impact of the socio-political context on community health nurses’ work and mothers’ daily lives.

**Conclusion:**

Findings offer insights about the potential and limitations of tailored nurse home visiting programs for refugee families, emphasizing the positive impact on mental health. However, challenges such as assimilation pressures, unwelcoming immigration policies, and discrimination may hinder program effectiveness.

**Supplementary Information:**

The online version contains supplementary material available at 10.1007/s00737-024-01529-y.

## Introduction

By the end of 2018, there were 2.4 million refugees in the European Union, half of whom women and girls, many in their prime childbearing years (UNHCR 2018). Refugee mothers often face multiple challenges: low incomes, linguistic barriers, uncertain residency, restricted healthcare access, and discrimination (Castaner et al. 2021; Stevenson et al. 2023; Niner et al. 2013). Further hardships emerge from disrupted social support networks, fostering feelings of loneliness and isolation (Ganann et al. 2020; Wachter et al. 2022). These strains, coupled with exposure to trauma during migration, manifest in the psychosocial well-being of refugee mothers: a recent meta-analysis showed a perinatal depression prevalence of 33.5% (Stevenson et al. 2023), double the rate of the non-migrant population. It is therefore crucial to develop maternal care models to bolster the psychosocial well-being of refugee mothers during the perinatal period (Balaam et al. 2021). Although there are various models and definitions of psychosocial well-being, it is generally recognized as a dynamic state of overall health, encompassing both psychological (e.g., self-acceptance, purpose, life satisfaction, autonomy, personal growth, and the ability to manage stress) and social (e.g., connectedness, sense of belonging, and positive social interactions) dimensions of a person’s life (Kumar et al. 2020; Burns 2017). This state reflects both intrapersonal and interpersonal functioning (Lent 2004; Silva and Pereira 2023).

This study delves into an add-on nurse home visiting (NHV) program with refugee mothers in Denmark, exploring their experiences and the program’s impact on their psychosocial well-being after childbirth. NHV programs stand as effective public health interventions to reduce health inequities and social inequality (Duffee et al. 2017). NHV has exhibited positive impacts on parental responsiveness, infant survival (Wüst 2012), child development (Henwood et al. 2020; Peacock et al. 2013; Richter et al. 2017), and maternal mental health (Goldfeld et al. 2021). There is robust evidence demonstrating the positive impacts of the Nurse-Family Partnership, an intensive prenatal and postnatal home visitation program for low-income first-time mothers, on maternal and child health (Miller 2015). However, little is known about how nurse home visiting can support the well-being of refugee mothers and how both mothers and healthcare providers perceive their participation in such programs (Molloy et al. 2021).

Clarke (2021) revealed the perceptions of refugee mothers regarding home visitors as being particularly instrumental in facilitating connections with the healthcare system. However, mothers expressed a desire for more support that addressed their roles as mothers and not solely the health of their children (Clarke 2021). Likewise, a UK study highlighted how home visitors had to prioritize essential physiological and safety needs, such as secure housing, as well as the needs of infants over those of mothers (Drennan and Joseph 2005).

**Fig. 1:**
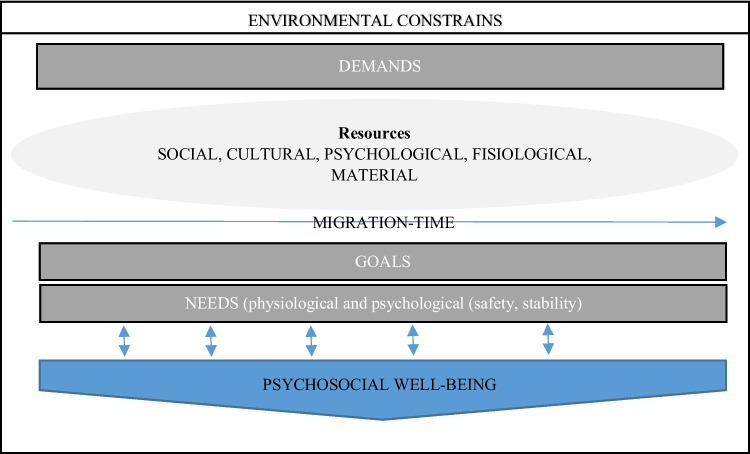
Graphic representation of the *Resource-Base Model* (RBM) of refugee adaptation (Ryan et al. 2008). This is a simplification of the model to highlight how concepts and relationships between them have informed our study

Through interviews with refugee mothers and community health nurses (CHNs), we zoom into a specific nurse home visiting program in Denmark to uncover insights into the *‘CHNs integration’ (*in Danish’ *Sundhedsplejersker styrker integration (SSI)* program’s effects and tensions. We ask refugee mothers about their experiences of participating in *‘CHNs integration’* and whether and how the program enable their psychosocial well-being in the transition to motherhood in Denmark. To do so we draw on the *Resource-Base Model* (RBM) of refugee adaptation (Ryan et al. 2008), which emphasizes how refugees’ psychosocial well-being hinges on how changes in available resources enable them to meet their needs, long-term goals and aspirations, and internal and external demands. Access to resources is influenced by the socio-political context, which can enable or constrain them. For example, temporary forms of residence can hinder refugees’ ability to find stable employment and restore material resources, leading to a negative spiral of *resource loss* and thus poor well-being. In contrast, the socio-political context can facilitate refugees’ well-being by providing holistic approaches that recognize refugees’ agency in defining their needs and goals (Chase & Rousseau, 2018). Using RBM as a theoretical lens, as depicted in Fig. 1, allows us to examine how refugee mothers perceive changes in resources from participating in the home visiting program. It also helps us explore how these changes relate to their needs, goals, and societal demands, and how they contribute to their psychosocial well-being. At the same time, this approach remains mindful of the structural tensions that may arise due to the socio-political context of the program.

## Methods

### Program description

**Fig. 2:**
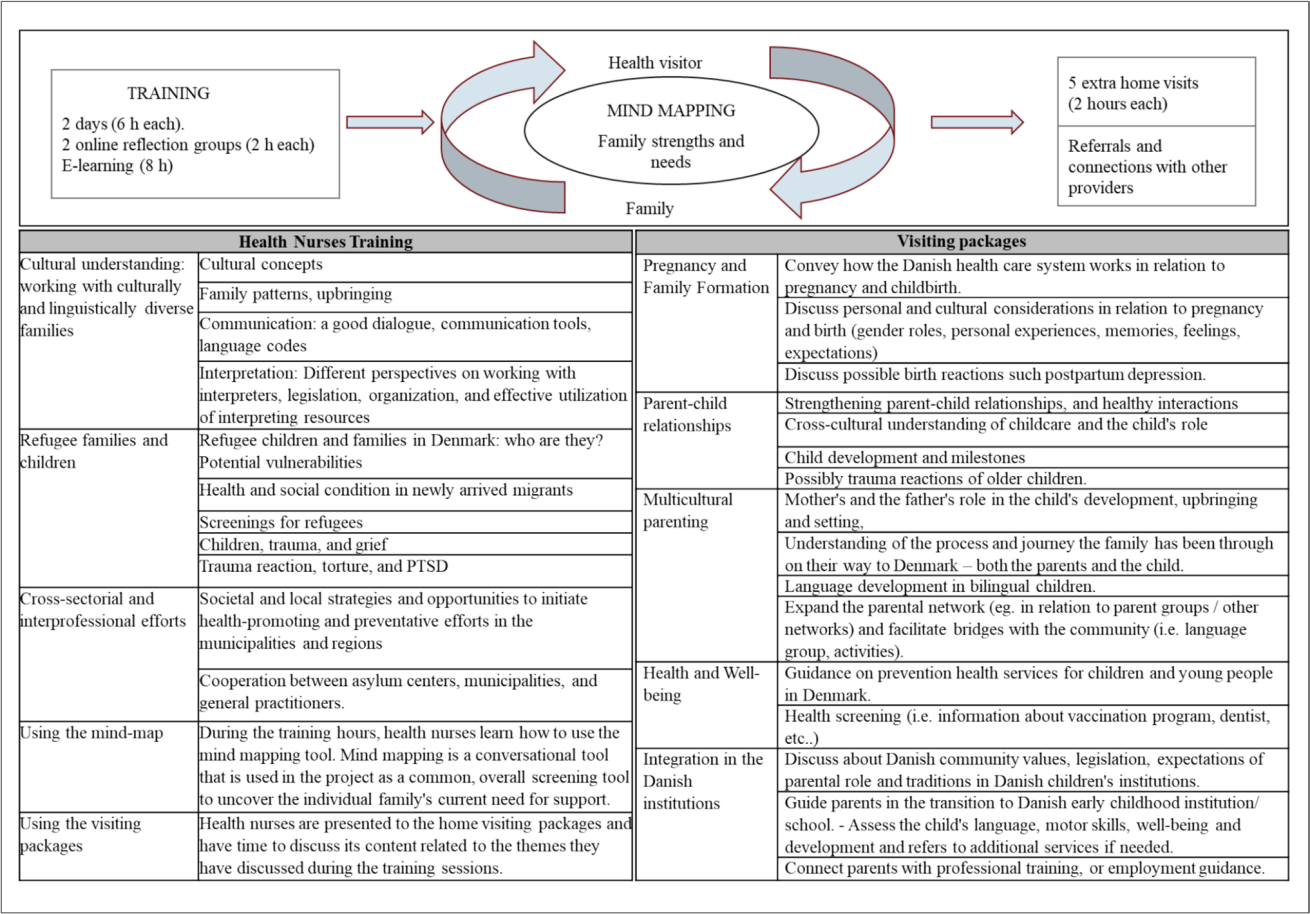
Program (Health Nurses Integration) description

SSI was implemented from 2017 to 2019 across 15 municipalities in Denmark for families with children under six. The program included five additional visits to the existing universal visiting program over one year. During the first visit, community health nurses (CHNs), supported by an interpreter, used a mind mapping exercise. Mind mapping, first described by Buzan and Buzan (Buzan and Buzan 2006), utilizes graphic representations to express major themes radiating from a central concept. This technique was designed to foster creative thinking, explanation and exploration of dilemmas and tensions. It has also been used in healthcare settings (Pringle 2019; Russell et al. 2020). In SSI, the CHN used mind mapping to understand the family’s background and needs. CHN asked questions like: ‘Who were you back home? What are the good things in your life now? What do you dream about? Is there anything challenging in your life that we can work on together?’ and, together with the families, recorded details about family members and areas where support was needed. This helped guide the selection of visit packages, which could be adjusted based on ongoing discussions. Subsequent visits focused on five broad themes: pregnancy, parent-child relationships, multicultural parenting, health, and integration (see Fig. 2). CHNs also connected families with other services. Although the program concluded after a year, some families continued to reach out to CHNs with questions about their children’s development and local resources. See appendices for further details.

### Study design and participants

The ethics committee at Copenhagen University approved the study, which began in September 2020. This study was independent of the SSI internal evaluation (Ramboll 2020). To provide a holistic perspective on the intervention, we included both community health nurses (CHNs) and refugee mothers. While the CHNs shared their views on the intervention’s underlying assumptions, goals, and their experiences with implementation, the mothers provided firsthand accounts of their care experiences and the impact of the intervention on their daily lives.

We employed a purposive sampling strategy to select four municipalities, where we interviewed eleven CHNs and nine refugee mothers. Initially, we planned to conduct group interviews with CHNs in each municipality. As a result, we conducted three group interviews with four, three, and three CHNs in each group, respectively. In the fourth municipality, however, only one nurse was available to participate. The purpose of the group interviews was to foster discussion among the nurses about their experiences and practices.

In contrast, we conducted individual interviews with the nine refugee mothers to provide a safe space to share their personal experiences. Our study focused specifically on mothers in the perinatal period. Therefore, we invited only women who had enrolled in the program either toward the end of their pregnancy or immediately after giving birth, and who were 18 years of age or older. Data was collected from September to December 2020.

All CHNs were part of SSI since the inception of the program, had at least five years of experience, and were Danish born. All nine mothers participated in SSI in 2019 and had refugee backgrounds. Mothers were from Syria (5), Eritrea (2), Iran (1), and Uganda (1), were between 23 and 37 years old, had between one to three children, and had lived in Denmark between one and six years. Five mothers had their first child in Denmark, seven lived with their partners and two were single mothers. Only one mother could communicate fluently in Danish.

### Data tools and analysis

Women’s interview guide aimed to gather participants’ experiences with SSI in the context of migration and becoming a mother in Denmark. The interview for CHNs focused on their experiences participating in SSI, challenges, and perceived benefits for women (see Appendix).

All participants could choose to be interviewed in their preferred language. Two women chose to be interviewed in English, one in Danish, one in Farsi, four in Arabic, and one in Tigrinya. In addition, two women requested to do the interviews with their husbands present. Group interviews and individual interviews with CHNs were conducted in Danish (3) and English (2). The first author participated in all interviews but performed only the ones in English. A Master student conducted the other interviews in Danish. Interviews in Farsi, Arabic, and Tigrinya were conducted with a bilingual interpreter. Due to Covid-19 restrictions, we did eight of the nine interviews with women and three of the five interviews with the group of CHNs using videoconference. Interviews were digitally audio recorded, then transcribed to English (see Appendix).

The data were analyzed in Nvivo using thematic analysis based on Braun and Clark (Clarke and Braun 2017). We followed five analytic steps: (1) becoming familiar with the data, (2) generating codes, (3) generating themes, (4) defining and naming themes, and (5) finding examples. The first author (MM) coded the data from mothers, while the second author (VP) coded data from community health nurses (CHNs). MM then reanalyzed both data sources to identify areas of convergence and divergence, ensuring distinct perspectives were acknowledged and highlighting overarching patterns. Using our theoretical framework and the concepts of resources, goals, and demands, MM identified twenty-seven basic themes reflecting the experiences of both mothers and CHNs (see Table 1).

**Table 1:** From basic themes to organizing themes

Basic themes		Organizing themes	
Demands to transition from community to individual forms of raising children		Negotiating parenting norms and gaining confidence through parenting resources	
Shared goal to give children a good future build trust			
CHN become families anchor in Denmark			
Mothers gain confidence through CHN guidance about food, sleep, development			
Balance between ‘Danish norms’ about upbringing and respect for families’ beliefs and traditions builds trust			
Using new parenting resources requires openness and negotiation			
Tensions of paternalism and assimilation pressures			
Integration pressure creates stress		Finding emotional support to cope with integration pressures	
Fear about the future due to uncertainty			
Mind mapping opens up a safe space to share			
Dialog about needs and goals brings recognition			
Sharing parts of own life brings emotional relief			
Reflecting on new possible gender norms fosters agency			
Mothers experience limited social networks		Expanding social resources	
Mothers feel doubts and insecurity about participation in mother group			
CNH encourage mothers to participate by empowering them			
Bridges to mother groups create new connections			
Bridges to mother groups help learning about Danish culture			
Mother groups foster friendship and social support			
when mothers have very limited social networks			
Mothers have agency to mobilize social connections autonomously			
Language barriers and lack of information lead to confusion navigating public services			
Discriminatory encounters in the welfare system		Building bridges with welfare services	
CHNs offer hand-holding and practical support based on families’ needs			
CHNs advocate for families ‘needs in other state institutions			
Handholding reduces stress of navigating a new system (healthcare, education, library)			
Encouragement and role-modelling in using services fosters autonomy			
Need for longer-term engagement and support to cope with structural pressures			

In collaboration with the last author (SFV), these codes were clustered into four main themes that captured the voices of both groups. To validate the findings, MM conducted two additional group interviews with seven of the eleven CHNs and discussed analytical ideas with other researchers in migration and maternal health. After presenting the results to various academic audiences, MM reviewed the data for evidence that could challenge the identified themes, ensuring the complexity of participants’ narratives was represented.

The main themes (see Table 1) serve as subheadings in the findings below, illustrating how the resources activated in interactions between health visitors and refugee families help refugee women navigate life in Denmark and support their psychosocial well-being.

## Findings

### Negotiating parenting norms and gaining confidence through parenting resources

Mothers experienced a transition from a community approach to raising children to more individualized forms of mothering in Denmark, where parents are considered *the* caregivers and are expected to make independent decisions about the pregnancy and their babies. Particularly first-time mothers experienced challenges navigating the demands of parenting in Denmark. ‘*I had a lot of worries. I didn’t have somebody to help me*,* and I couldn’t speak the language. I didn’t know where the doctor was and how you raise a child.*’ (M2). In this transition, CHNs played an important role in strengthening women’s agency and resourcefulness.

*In my country the family does everything. Here when you have a child you do it yourself. But I say to myself*,* I can do it*,* I can do it. When I don’t understand something*,* I call Maria (the CHN). (M1)*

CHNs were described as part of ‘us’, the refugee family, but also part of ‘them’, Danish society. As Naila put it, ‘…*she (CHN) has become a part of us*,* this is something good (M6)’.* From a resource-based perspective, this relationship was valuable because mothers and CHNs shared the same aspirations–providing a good future for the children. Women described CHNs as ‘*baby experts’* and that meant they could be trusted to talk about parenting. Through the relationship with the CHN, mothers gained knowledge and practical guidance about infant healthy eating, sleeping routines, and a child’s health or developmental expectations, contributing to their increased confidence in raising their children in Denmark. CHNs often became a substitute for the female kindship they missed and allowed them to maintain some collectivistic disposition.

*‘She (CHN) is my friend now. When I have problems*,* she tells me ‘call me if you need anything about Ana (the child)’… I was very happy that now my kid is in good hands’ (M1).*

Mothers also highlighted CHNs’ role in resolving tensions arising from different familiar parenting practices and parenting Danish norms, such as early introduction of solid foods, encouraging mobility from a young age, letting babies sleep outside in prams or bringing babies to daycare at ten months.

*I was frustrated. My son has pain in his stomach*,* I want medicine*,* here in the pharmacy they didn’t give me medicine*,* she (CHN) said to me*,* “they (parents)*,* here*,* they don’t use medication a lot” … She (CHN) said*,* “with basic movement you can (help) relax your child”. Then it means*,* there are no reason for the tension and frustration*,* it means*,* things are simple. (M5)*

When mothers had to negotiate between the ways of caring for children that were known to them with Danish normative parenting, the CHNs offered families an insider view into new parenting norms. ‘*How to raise a child is different in Denmark compared to Iran. She taught us the Danish way of raising children because my child will live in Denmark*,* so that was a big help” (M4)*. From a resource-based perspective, mothers gained new parenting resources in dialogue with CHNs.

However, the idea that there is a ‘Danish way’ of parenting may also become problematic. One mother described how other mothers in her community found the support unnecessary. ‘*There are some of my friends that were annoyed by the repeated visits. They said*,* we are also mothers and have raised [children]*,* so we know*,* this is not necessary (M4)’*, suggesting how normative motherhood (O’Reilly 2023) can turn from a helpful resource to an oppressive structure and create tensions between families and CHNs that represent the welfare state.

From the nurses’ perspective, discussing Danish parenting norms was crucial to ease transitions into Danish society and educational institutions. However, CHNs recognized how that must entail a challenging dialog and negotiation where CHNs fostered the value of parenting resources and cultural background and reflected about their own position. ‘*We (CHN) have to take a little step back from our own culture and listen to the culture that we meet (CHN4)’.*

### Finding emotional support to cope with integration pressures

Most mothers found emotional support in their conversations with CHNs. They offered families a space to share and helped them find additional support if they needed. The mind map activity facilitated this process. Mothers described how the experience of drawing, and visualizing their past and present life, their strengths and needs, helped them give meaning to some of the experiences they had and how they felt.

*‘Imagine doing and drawing your whole life on a paper*,* that’s something that relieved me a lot.’ (M4).*

This was particularly important for women who were distressed like one mother said:

‘*my psyche was very tired…from the weather*,* the alienation*,* being far from home*,* being a stranger in a country*,* it was very hard.’* (M5). For her, the most important aspect of the program was the emotional support. ‘*I felt that she helped me more rather than the children…I really relaxed when she talked to me*,* her concern about me*,* I really relaxed.’ (*M5).

Mothers also experienced demands to learn Danish and enter the labor market. Such demands could become excessive and detrimental to well-being. ’*There is a system from above that says you have to go to work*,* you have to get an education*,* otherwise we will take your services. And there is a family that has a trauma*,* that make it really hard to live up to’ (CHN5).* CHNs had the double role of promoting integration while caring for the family’s well-being. CHNs tried to turn the ‘pressure’ to integrate into encouragement to explore educational and job opportunities while advocating for families in front of integration officials. Such emotional support encouraged some mothers to challenge gender norms and pursue new opportunities outside the home.

*I told her ‘I am feeling this way’ (feeling sad). She (CHN) tried to help me. All my thought was that I had to stay within the limits of the house. I am a mother that has children (four). I should stay at home. I should take care of these children. That was my thoughts. But after I sat with her…I can also go out*,* I can also study*,* I can also work*,* I can do a lot of things…I started thinking more*,* I was more relieved*,* It is really possible that I can also do that (M4).*

CHNs experienced this dialogue as a balancing act, where they were curious about families’ background and offered emotional support while discussing what CHNs described as ‘Danish values’ important for integration (i.e. gender equality).

However, the emotional support, the encouragement and recognition, could not help mothers cope with the uncertainty of their temporary permits and public discourses against refugees. At the time of the interviews, the Danish government had started revoking some refugees’ residency permits. All women expressed their fear and worry for their children’s future.

*I think my boys have a good future here (in Denmark) but I am also worried about what is happening*,* seeing that they want to send some Syrians back. And that really worries me (M7).*

When women started to imagine a future in Denmark, anti-immigration policies reminded them about the uncertainty of their legal status, which made them feel unwelcomed, and threaten their well-being. ‘*When they (government) speak that refugees shall return. I don’t feel like there is future’ (M5).*

### Expanding social resources

CHNs facilitated mother groups and encouraged mothers to participate in local activities such as Danish language groups. Participating in these groups created at first some insecurities among mothers. ‘*I hesitated*,* the language*,* I don’t know it.I didn’t have any motivation to go’ (M5).* From CHNs’ perspective, expanding mothers’ social networks was a way of supporting their mental health and their integration into Danish society ‘In *relation to mental health*,* this is exactly where we can help to create a network…The network is incredibly important*,* to get out among others and see some role models (CHN6)’*.

From a resource-based model perspective, by framing these interactions as a place where refugee mothers could exchange their experiences and knowledge, CHNs contributed to reinforcing mothers’ psychological resources of self-efficacy and helped mothers to gain new social resources.

*She encouraged me*,* she said ‘you should try*,* and see*,* you will be better …language isn’t important*,* there is a lot one can do*,* with signs… Its right*,* they might be educated*,* but you also have experience…they will also be benefited from you*,* and you will benefit from them"…And it was a really beautiful experience*,* There were difficulties in the language…But it passed*,* we understood each other (M4).*

Connecting with other mothers enabled them to create new bonds *‘I can’t say that they replaced my mother and mother-in-law*,* but it was a replacement*,* a replacement that I was very comfortable with’*, and create friendships and a sense of community.

CHNs recognized that creating these opportunities to expand mothers’ social networks was challenging, for practical reasons (i.e. lack of transport), and a lack of a shared language or experiences. Few mothers also discussed how they had created their own networks (family members that migrated before, or mothers they meet at the asylum center). Therefore, ‘prescribing’ mother groups was not suitable. Instead, understanding mothers’ needs for new *social resources* and actively facilitating such opportunities, particularly when mothers experienced social isolation, was considered more effective.

### Building bridges with welfare state services

From a RBM perspective, mothers shared a *demand overload* as they lacked cultural resources (e.g. Danish language and knowledge about welfare services) to navigate welfare state services, namely healthcare and early childhood services, that were not fully adapted to meet the needs of newly arrived refugee families. These challenges were compounded by the rigid rules of the integration system.

“*They [job centers] have these rules they follow*,* and if you have been absent four times*,* they take your subsidies*,* and then we push them [refugee families] further and further out*,* and they [refugee families] cannot always do that. They do not have the resources to tell what is important and why it has been that way. (CHN 5)*

When parents had to contact the healthcare system, sign up their children for nursery, or navigate the demands from integration services, CHNs offered practical resources. They went to the nursery with families, assisted with housing support applications, or called their doctor to ensure their needs were understood and they could receive care promptly‘*I didn’t have the knowledge about signing up*,* and when I had a meeting with the daycare*,* she (nurse) came with me. It was nice having someone caring. (M3)’*. Receiving such support made families feel connected and cared for by the CHN.

CHNs also advocated for families, particularly when facing multiple adversities, such as trauma, low literacy, or disability, attempting to convince integration services of the harmful effects of overburdening families with strict rules and regulations.

*CHN4: we could use that collaboration with the job center and the integration department*,* and the fact that if there was a problem…was it you who had a family*,* where we redeemed the mother from not doing an internship or language school for a period of time? (CHN4)*

*CHN3: It was me yes. A mother who had just lost her husband. They had recently arrived in Denmark*,* and he passed away from a very serious illness. She was in a new country*,* alone*,* with her twin children. She had come from another municipality*,* and in this one*,* they told her: “You have to attend language school*,* you have to do an internship*,* and the children must be cared for outside the home.” When I visited her*,* she was completely overwhelmed. She looked terrible*,* so I had to contact various agencies*,* arrange a meeting at the kindergarten*,* call the job center*,* and consult my own doctor—basically*,* coordinate everything to create a unified plan. Eventually*,* she was exempted from both the internship and some of the other demands*,* and she started attending language school. She began to improve*,* and I see her often now. She lives on a road I frequently drive along*,* so we wave at each other. She looks completely different these days.*

CHNs leveraged their positions to negotiate with institutions responsible for family integration. The project empowered them to challenge rigid integration plans set by job centers, language schools, and case managers, which often overlooked the specific needs of families. While CHNs were aware of integration rules and their role was to support integration into Danish society, they based their decisions on discussions with families and the relationships they developed over time. Mothers noted that CHNs understood both their needs and preferences and the workings of Danish institutions, allowing them to act in the families’ best interests: ‘*she helped me to find the best daycare for my child (M3)*’.

CHNs went beyond their usual roles, helping mothers rehearse phone conversations with doctors, which equipped them with the skills to handle similar situations independently after the CHNs had left. By gaining social and cultural resources through their relationships with CHNs, mothers felt less stressed navigating unfamiliar systems and became more confident in advocating for themselves. One mother told her CHN,

“*You have given me faith that I can do things myself. Everyone wants us to succeed*.”

However, CHNs acknowledged that learning to navigate these systems required time—often beyond the duration of the program—and a positive attitude from professionals interacting with refugee families. They reflected on the limitations of their roles within a broader social system that remained challenging for refugee mothers, where they continued to experience discrimination. The following exchange between two CHNs highlights the complexities of the system and their frustrations in trying to assist refugee mothers:

*CHN 1: The experience of the distrust to the system and the fact that we also are a part of the system*,* we also hear that they have distrust elsewhere. Job center*,* the bank…*.

*CHN 2: I sometimes get asked “it’s because I’m dark that they do not want to talk to me”*,* “You must help me then” and that feeling of not being good enough and that you are not welcome… we can not take their background away from them…Integration is a difficult size … we’ve got our eyes on it too. Not that it should sound negative but it is true that we have seen that it is a wide size and much wider that our program....*

*CHN 3: You have to help them with everything possible with these contexts and when I meet them again a few years later*,* they say “we are in the same position”. During SSI we collaborated a lot with integration consultants. We could give them some tasks*,* and it was really good*,* they were really happy that we could have this collaboration. We also had some meetings together. But this is closed now.*

Overall, the SSI project gave CHNs the flexibility to engage with integration services, early childhood education centers, or doctors, advocating for refugee families. By helping families understand and engage with welfare services, CHNs guided them in overcoming barriers while nurturing their self-efficacy. However, concerns remain about the pressures to assimilate and experiences of discrimination and racism within and outside public institutions.

## Discussion

Our study, informed by the RBM of refugee adaptation, underscores the positive impact of a tailored home visiting program for refugee families. Specifically designed to support the well-being and integration of refugee families, this program expands mothers’ *resource pool (*Ryan et al. 2008), aiding them in navigating the demands of integration into Danish society and strengthening their psychosocial well-being. Our findings reveal that refugee mothers acquire parenting resources, emotional support, and opportunities to build social networks which enhances their self-confidence, autonomy, and reduces stress, while by focusing on their shared goal of providing a better future for their children. This aligns with studies exploring postnatal care models involving peers or bicultural workers (Paris 2008; Rogers et al. 2020, 2023).

Notably, our work emphasizes how refugee mothers can form meaningful bonds with home visitors who don’t share their cultural background and who represent a welfare state institution. By allowing time for relationship-building, flexibility in defining CHNs roles, balancing family’s needs and pressures to integrate, and having a shared focus on the baby’s well-being, trust is cultivated, enabling mothers to gain resources from this connection. This is aligned with Willey et al. (2018), who argues that nurses require more time and flexibility to build relationships with refugee families and develop intercultural communication skills and cultural self-awareness. Our findings emphasize the importance of understanding the “*gain of resources*” in the resource-based model (Ryan et al. 2008) within a relational context, where health and social care providers can create a safe space by listening and responding to the families’ needs and goals (Kassam and Marcellus 2022).

However, our research also highlights the impact of the broader socio-political context and assimilation notions on mothers’ ability to utilize resources for their aspirations. Mothers experience a dissonance between feeling cared for by CHN and feeling unwelcomed in Denmark and pressured by the Danish government. Recent shifts in Danish immigration policies, with weakened protection and stricter requirements to obtain permanent residency, intensify concerns voiced by women about the effects such policies have on themselves and their children.

Structural injustice theories (Young 2001; Powers and Faden 2019), allow us to critically analyze the constraints within which CHNs operate. Applying a structural injustice approach, which focuses on the way in which economic and social structures affect mental health and well-being rather than zooming on individuals and their conditions, allows us to see the limit of ‘the assistance’ the CHNs could offer refugee mothers.

Firstly, it can be argued that the support is founded on an ideology of integration, which runs the risk of increasing pressures to assimilate. Despite mothers being grateful to learn about Danish parenting norms and expectations, these approaches should critically re-assess and carefully revise static assumptions about ‘good Danish parenting’ that potentially dismiss refugee’s mothers parenting resources, values, and beliefs (Matthiesen and Society 2019). As some CHNs pointed out, negotiating parenting norms and what is best for the children requires a constant dialogue between families and CHNs, where CHNs dare to revise their own cultural assumptions about ‘good parenting’. Still, when there is pressure to assimilate (exerted by integration policies), refugee mothers may be confronted with a problematic trade-off which might lead to dismissing some of their native practices not necessarily because they are inferior but only because such practices will make integration harder.

Secondly, the CHNs create bridges between mothers, families, and other institutions, yet, developing knowledge and understanding about how welfare state institution work takes time and mental resources. The fact that refugee families experience discrimination and racism across other institutions is a reminder that promoting the psychosocial well-being of refugee mothers and their children must be a collective effort and that should extend beyond the period of early childhood.

In sum, we argue that there is a need to further examine the effectiveness of nurse-home visiting models such as the one we have studied to improve maternal mental health outcomes among refugees. More importantly, effectiveness studies must examine how social and structural determinants intersect with well-intentioned public health programs.

Our findings have several limitations. Since the study was not part of the original intervention design, we only accessed refugee women through the CHN, potentially introducing selection bias toward positive program experiences. Still, mothers reflected on broader community experiences, revealing tensions like paternalism and assimilation narratives. Future research should also examine families with minimal program engagement.

Additionally, our small sample of municipalities and participants limits generalizability. While mothers consistently benefited emotionally and socially from the program, further research should consider the impact of contextual factors like community resources and organizational factors of nurse visiting programs in diverse contexts and municipalities. Nevertheless, the use of the RBM enables theoretical abstraction, fostering relevance to other studies analysing motherhood in diaspora.

In conclusion, our findings underscore the importance of advocating for family-centered models that address the unique needs of refugee families in the broader community, social, and cultural context. Tools such as mind mapping may facilitate dialogue between families and providers, fostering a mutual understanding of the families’ goals and needs. This, in turn, can support CHNs in providing social, cultural, and emotional resources to refugee families. Such models can assist mothers in navigating parenting in a foreign country and promote their mental health. However, we must further unpack how underlying assimilation assumptions can create tensions and disengagement between welfare services offering postnatal care and refugee families. More participatory methods that engage communities in the co-design of interventions can foster models of postnatal care that are more inclusive and less shaped by normative assumptions around motherhood.

## Supplementary Information

Below is the link to the electronic supplementary material.

ESM1 (DOCX 248 KB)
