# Supplementary material for: Refugee mothers’ mental health in Denmark: possibilities and limits of home visiting programs
Source: Arch Womens Ment Health. 2024 Nov 19;28(2):245–55. doi: 10.1007/s00737-024-01529-y (PMC12018596; doi:10.1007/s00737-024-01529-y)
Supplement: Supplementary file 1 — (DOCX 248 KB) [file 737_2024_1529_MOESM1_ESM.docx]

**Appendix**

**Methods**

**CHNs Strengthen Integration**

The study was conducted in Denmark, where all municipalities offer a nurse home-visiting postpartum program to all families with newborn children. Approximately 97% of families accept this offer. CHNs visit the family on an average of four occasions: during the first or second-week post-birth, between 2 and 3 months, between 4 and 6 months, and between 8 and 11 months. CHNs assess the infant development, families well-being, and mother’s mental health status at each visit, following national guidelines. The evaluation is based on the mother’s view and the CHN’s clinical judgment.

In addition, CHN connect mothers in the same area to meet during their maternity leave. Mother groups are a regular offer that health nurses make to all women giving birth in Denmark. Health nurses find mothers in the same area and help them connect after giving birth to share their experiences and find social support. Sometimes health nurses try to connect migrant mothers, but this is not always possible. Mothers decide where and how often they meet while they are on maternity leave. These groups are central to the peer-to-peer postpartum care of women in Denmark.

Between 2014 and 2016, Denmark experienced a fourfold increase in asylum seeker applications. Approximately 33,300 asylum seekers sought asylum in Denmark during that period, with 64% being granted refugee status. *CHNs integration* *(*in Danish' Sundhedsplejersker *styrker* integration *(SSI))* was developed in 2016 by the Association for Managers of Health Schemes for Children and Young People in Denmark (FALS) in collaboration with the VIA University College and Metropol vocational college*,* as an enhanced home-visiting program that targeted the needs of refugee families and prepared community CHNs (CHNs) for such a task. SSI targeted some of the challenges that prior work had identified (need for training in cultural competence, limited visiting time, and language barriers). In addition, it aimed to support the refugee families' integration into Danish society, their health, and well-being. The program was designed to enhance the universal Danish nurse home visiting program, which offers at least five visits in the first year of the child's life with the overall aim of promoting child health and development.

SSI was offered to newly arrived refugee families either expecting a child or having children under six years of age. The project consisted of three main elements that worked in synergy (see Figure 1). The first one was a competence development course for community CHNs. CHNs participated in a four-day training in cultural competencies to understand the role of culture in communication with refugee families and learn to use mind-mapping techniques to assess the families' needs. The course also included training to work with interpreters, as they were provided access to them. Before the program was implemented, interpreters were available but CHN experienced challenges to use them regularly and work with them. The second element of the project consisted of five extra visiting packages. These were long themed home visits (2 h), which the nurses delivered to each refugee family based on their specific needs identified in the mind mapping exercise. These five visits were added to the five existing visits that CHNs regularly provide. Last, CHNs were given more time and flexibility to connect families with Danish institutions (healthcare, school, integration services) and coordinate the care of refugee families.

An external evaluation of SSI, based on survey data from 100 nurses and qualitative interviews, showed that most experienced increased cultural sensitivity and a sharpened view of their coordinating role between the family and the surrounding providers (see link for the full report below). The report also concluded that the project had given the families more knowledge and the opportunity to use the Danish healthcare system. Based on data from the Danish national registries, children in the refugee families had more contact with the healthcare system at the end of the project compared to a control group of refugee children who had not been part of the project. However, no measurable change in the occurrence of preventive measures (engagement in social services) and parents' labor market participation was identified. The evaluation did not examine mothers' mental well-being concerning SSI.

SSI was implemented across 15 municipalities, out of 98 in Denmark, 2017-2019, with funding from a non-profit foundation. Efforts were made to do visits with mothers and fathers. However, in practice, fathers did not attend all visits due to work obligations.

The summarized content of health nurses training and visiting packages is described in Figure 1. In a first visit, CHN used a mind mapping exercise together with an interpreter, to understand the refuge family's story, strengths, and needs. Based on this first visit, five additional home visits follow organized around five general themes. In addition, the CHN has the role to connect families with other services (i.e. job centers, language schools, or general practitioners) and coordinate the care of families with their permission.

**SSI internal report:**

<https://www.via.dk/efter-og-videreuddannelse/sundhed-omsorg/sundhedsplejersker-styrker-integration>


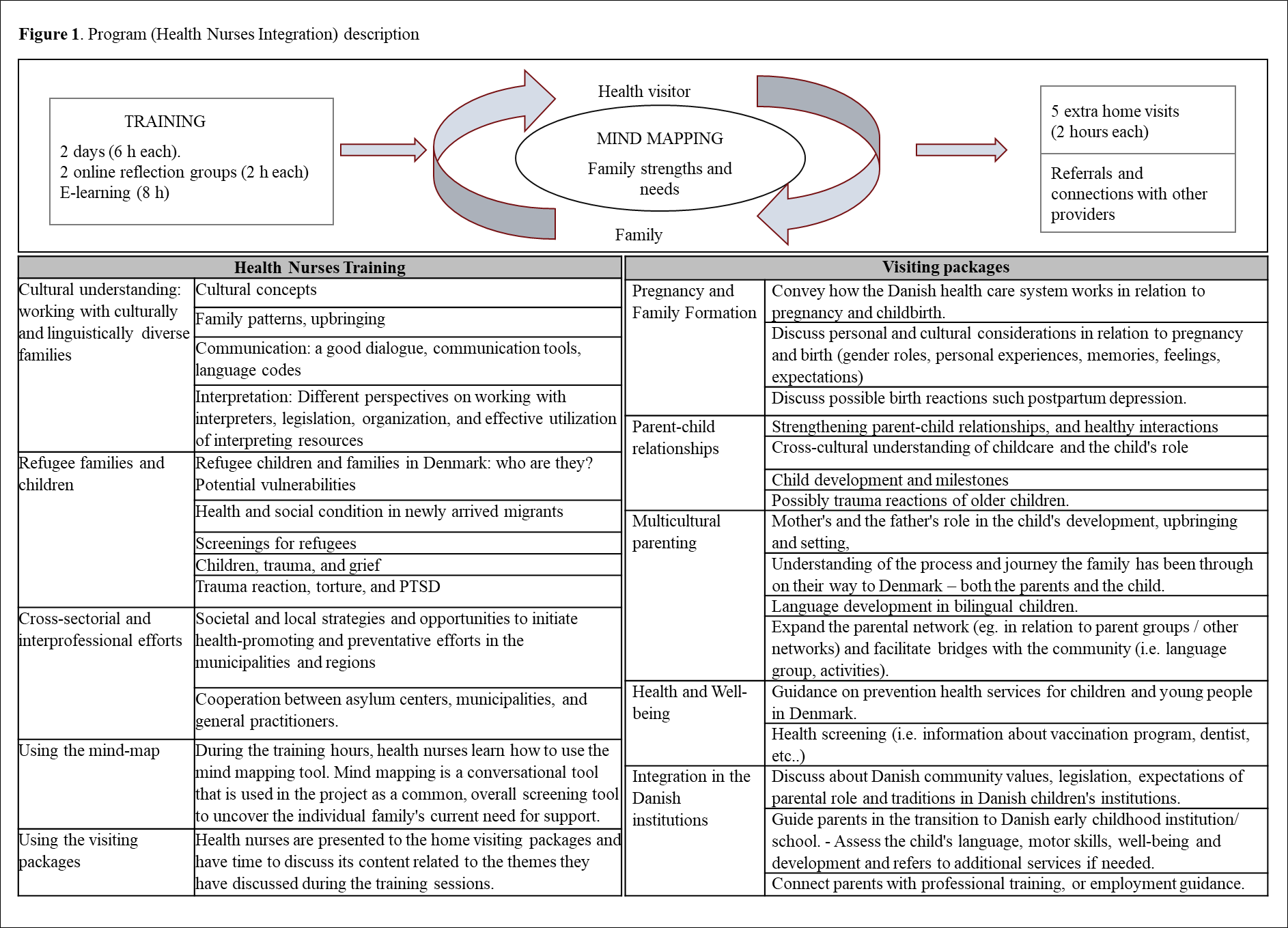


**Data collection**

All participants could choose to be interviewed in their preferred language. Two women chose to be interviewed in English, one in Danish, one in Farsi, four in Arabic, and one in Tigrinya. Group interviews and individual interviews with health nurses were conducted in Danish (3) and English (2). The first author participated in all interviews but performed only the ones in English. A Master student involved in the study completed the other group interviews in Danish. Interviews in Farsi, Arabic, and Tigrinya were conducted with a bilingual interpreter. Two interpreters were medical students, and one was a professional interpreter working with the municipality. Interviews were digitally audio recorded, then transcribed to the original language and translated to English by bilingual interpreters.

**Interviews**

**Interview Guide for families**

**Section 1: Timeline**

I would like to start making a timeline about your life since you moved to Denmark to understand better your experience with SSI. For example, if I did a timeline about my live since I moved I would … (show timeline with date when I moved, with whom, date when I start working, when children started school, changes house)

Show line, then ask:

- Could you mark the year when you arrived to Denmark?
- Where did you come from?
- Who did you come with?
- Where have you lived during this time? Have you moved since you arrived to Denmark?
- When did you participated in SSI? When did you saw your health visitor for the last time?
- Since you came to Denmark up to now (show line), could you mark any important events in your life?

**Section 2: Families’ life before the intervention started**

1. I would like to start asking you about your life right before you took part of SSI (show timeline)? Tell me how you and your family were doing back then.
   - How did an ordinary day look for the family?
   - What were you happy about / satisfied with?
   - Did anything worry you? / struggle with? What made you feel like that?
2. Now let me ask you about how you felt when you became pregnant and you child was born in Denmark
   - How did you feel during your pregnancy?
   - How did you feel when your child was born?
   - Why (what made you feel like that?)
3. Some women feel a bit sad, worried, and tired when they are pregnant or when the child is born. This is very common.
   - Did you ever feel like that?
   - Why/What made you feel like that?
   - Was there something that made change how you felt?
4. Sometimes refugees may feel sad or have ‘bad’ memories because of what they experienced in their country or their life in a new country.
   - Did you ever feel like that?
   - How that affected your life?
   - And your relationship with your child?

**Section 3: Families’ experience of SSI and perception of impact**

Now I would ask you some questions about your experiences with your health visitor

1. When the Health visitor from SSI called you and asked you if she could visit you and your family, what were your expectations?
2. Tell me more about what you remember from the first time you met your health visitor from SSI (show timeline)?
3. Can you give me examples of things you talked about or discussed with your HV? How was it for you to discuss those issues? How did you feel about it? What did you think about it?
   - Probe: parenting, school, Danish culture, mental health?
   - Did you learn something new? Can you give me an example?
4. How do you think the program has supported you and your family?
   - Can you give me three detailed examples of how the program helped you and your family?
   - Did you change the way you do things in your family? Can you give me an example?
5. Did the CHN go with you to the kindergarten, or the doctor, or the municipality?
   - How that made you feel?
   - How was that helpful?
6. Did you talk with your health visitor about being a parent here in Denmark?
   - What do you remember?
   - How that made you feel?
   - How was that helpful?
7. Did the CHN put you in contact with other families?
   - Could you tell me more about that? How was that experience?
8. Did you ever talk with your HV about how you were feeling after having your baby (reference question 6: being sad/references to trauma)?
   - How that made you feel?
   - How was sharing this with your health visitor?
   - If you did not talk about that with her, why you did not want to share it with the CHN?
   - Did you ever feel you needed additional support? (for example, see a doctor or someone also to talk about your feelings? Did you get that support? How?

Now I have two more question for you:

1. What did you like the most about your meetings with the health visitor? Why?
2. What did you like the least about your meetings with the health visitor?
   - Was there anything challenging about your meeting with the health visitor?
   - Where there times when you felt uncomfortable? (why)
   - Is there something you would have liked from the HV and didn’t get?
   - What would you change?

**Interview Guide for Health visitors**

**Section 1: Personal Motivations to participate in SSI**

1. How did you get involved in the SSI project? What motivated you to take part of it?

**Section 2: Health visitor characteristics/experience and work “context”**

1. For how long have you been participating in SSI?
2. How many other health visitors in your Kommune are part of SSI?
3. What was your experience working with refugee families before implementing SSI?
4. Before SSI, what was your job? For how long have you worked as a health visitor?
5. Did you ever get specialized training in mental health?

**Section 3: Health visitor experiences with SSI**

1. Overall, how would you describe your experience with SSI?
2. Tell me about how SSI is implemented in your Kommune?
   1. Tell me in detail what you do the first time you meet with a family?
   2. Tell me what you do after the first meeting.
3. How do you build a relationship with the family?
4. What have been the challenges in implementing SSI?
   1. Will you share with me one or two examples of challenges implementing SSI?
   2. What makes your work difficult?
5. How families have responded to the program.
   1. Will you share with med one or two examples of how families have responded to SSI?

**Section 4: Health visitors’ perception of SSI impact**

1. How SSI benefits refugee families?
   1. Can you give me two detailed examples?
   2. What makes it ”work”?
2. Can you tell me about a time when you feel SSI did not work well or was not enough?
   1. Why the program did not ‘work’?

**Section 5: Supporting mental health of refugee parents through SSI**

Now I would like to discuss more about how SSI support the mental well-being of refugee parents.

1. What emotional or mental health issues you have observed working with refugee parents.
   1. Probe about postpartum depression
2. In your experience, what are the things that affect refugee women mental health?
   1. Can you give me an example?
3. Can you give me examples of how you address some of these issues in your work?
4. When do you decide to refer parents to receive additional support for the mental well-being?
   1. How do you do it?
   2. What has been your experience referring them?
   3. How do they respond?
5. Has the training received through SSI changed the way you support the mental well-being of refugee parents? How?
   1. What do you do different now?
6. What else you think is needed to support the mental well-being of refugee parents after having a child?
